# Supplementary material for: MetaRibo-Seq measures translation in microbiomes
Source: Nat Commun. 2020 Jun 29;11:3268. doi: 10.1038/s41467-020-17081-z (PMC7324362; doi:10.1038/s41467-020-17081-z)
Supplement: Supplementary file 10 — Supplementary Data 7 [file 41467_2020_17081_MOESM10_ESM.zip › File2/Confidence_High_Taxonomy/356727_out.krona.html]

Javascript must be enabled to view this page.

members
magnitude
magnitudeUnassigned
count
unassigned
taxon
rank

356727\_out

121

2
superkingdom
121

phylum
121
976

200643
class
121

121
order
171549

family
120
1853231

genus
120
283168

1897010

SRS046717\_contig\_number\_5029SRS063985\_contig\_number\_19453
2
species

28118
116

SRS011084\_contig\_number\_15915SRS011134\_contig\_number\_38480SRS011239\_contig\_number\_contig-100\_224.157579SRS012849\_contig\_number\_contig-100\_604.190126SRS012969\_contig\_number\_35447SRS013098\_contig\_number\_36318SRS014459\_contig\_number\_22621SRS015431\_contig\_number\_69130SRS015579\_contig\_number\_20317SRS015663\_contig\_number\_24739SRS015960\_contig\_number\_18017SRS016095\_contig\_number\_960SRS016335\_contig\_number\_11229SRS016954\_contig\_number\_27146SRS017191\_contig\_number\_15965SRS017247\_contig\_number\_8507SRS017433\_contig\_number\_5364SRS017521\_contig\_number\_20175SRS017821\_contig\_number\_799SRS018133\_contig\_number\_13693SRS018623\_contig\_number\_19407SRS018836\_contig\_number\_12685SRS018888\_contig\_number\_8252SRS019068\_contig\_number\_95774SRS019161\_contig\_number\_41838SRS019397\_contig\_number\_8335SRS019601\_contig\_number\_contig-100\_193.157166SRS019968\_contig\_number\_4738SRS020233\_contig\_number\_27060SRS020271\_contig\_number\_contig-100\_676.677SRS020328\_contig\_number\_30290SRS020394\_contig\_number\_contig-100\_719.85901SRS021219\_contig\_number\_7340SRS021484\_contig\_number\_28878SRS022137\_contig\_number\_20684SRS022524\_contig\_number\_3038SRS022609\_contig\_number\_50221SRS023526\_contig\_number\_37413SRS024075\_contig\_number\_5946SRS024435\_contig\_number\_6118SRS024492\_contig\_number\_8631SRS043001\_contig\_number\_contig-100\_268.62256SRS043701\_contig\_number\_11673SRS043768\_contig\_number\_27076SRS043841\_contig\_number\_10807SRS044535\_contig\_number\_4175SRS046068\_contig\_number\_751SRS046369\_contig\_number\_10054SRS047014\_contig\_number\_29611SRS047433\_contig\_number\_18850SRS048164\_contig\_number\_contig-100\_58.155543SRS049402\_contig\_number\_16690SRS049446\_contig\_number\_11005SRS049773\_contig\_number\_36420SRS049900\_contig\_number\_19971SRS049995\_contig\_number\_20780SRS050026\_contig\_number\_5577SRS050925\_contig\_number\_34854SRS051031\_contig\_number\_contig-100\_237.178141SRS051610\_contig\_number\_9612SRS051882\_contig\_number\_19558SRS052697\_contig\_number\_contig-100\_134.236481SRS053214\_contig\_number\_21414SRS053398\_contig\_number\_contig-100\_779.154673SRS054352\_contig\_number\_13770SRS054905\_contig\_number\_12345SRS055982\_contig\_number\_1803SRS056273\_contig\_number\_11147SRS058770\_contig\_number\_27187SRS063040\_contig\_number\_29869SRS064557\_contig\_number\_22549SRS064757\_contig\_number\_23990SRS075078\_contig\_number\_28069SRS075773\_contig\_number\_5324SRS076804\_contig\_number\_20640SRS077127\_contig\_number\_contig-100\_175.116121SRS077194\_contig\_number\_13627SRS077231\_contig\_number\_19360SRS077392\_contig\_number\_26844SRS077730\_contig\_number\_3505SRS077849\_contig\_number\_4671SRS078176\_contig\_number\_20704SRS078242\_contig\_number\_28211SRS078665\_contig\_number\_10037SRS097958\_contig\_number\_1719SRS098073\_contig\_number\_6668SRS098514\_contig\_number\_contig-100\_61.61SRS100021\_contig\_number\_47SRS101433\_contig\_number\_14344SRS103987\_contig\_number\_23038SRS1041118\_contig\_number\_2176SRS1041134\_contig\_number\_2459SRS104311\_contig\_number\_45447SRS104636\_contig\_number\_30622SRS1054716\_contig\_number\_6442SRS1055083\_contig\_number\_contig-100\_634.35959SRS140492\_contig\_number\_31299SRS140513\_contig\_number\_5577SRS142503\_contig\_number\_14186SRS143876\_contig\_number\_39387SRS143895\_contig\_number\_33303SRS144362\_contig\_number\_25018SRS146764\_contig\_number\_29204SRS147139\_contig\_number\_14523SRS147271\_contig\_number\_26487SRS147557\_contig\_number\_33572SRS148159\_contig\_number\_55943SRS148196\_contig\_number\_42911SRS148721\_contig\_number\_45973SRS149181\_contig\_number\_30261SRS893170\_contig\_number\_207SRS893369\_contig\_number\_10258SRS893373\_contig\_number\_725SRS893378\_contig\_number\_19272SRS971275\_contig\_number\_39282SRS971276\_contig\_number\_26805
species

species
1

SRS015782\_contig\_number\_45359
1263090

1637502
species

SRS1055076\_contig\_number\_9513
1

family
1
815

816
1
genus

1

SRS144506\_contig\_number\_40884
species
821
